# Intermittently Administered Parathyroid Hormone [1–34] Promotes Tendon-Bone Healing in a Rat Model

**DOI:** 10.3390/ijms151017366

**Published:** 2014-09-29

**Authors:** Fanggang Bi, Zhongli Shi, Shuai Jiang, Peng Guo, Shigui Yan

**Affiliations:** Department of Orthopedic Surgery, the Second Affiliated Hospital, School of Medicine, Zhejiang University, No. 88, Jiefang Road, Hangzhou 310009, China; E-Mails: 163bfg@163.com (F.B.); zlshi_78@163.com (Z.S.); zjujs001@163.com (S.J.); pengguojx@sina.com (P.G.)

**Keywords:** parathyroid hormone, anterior cruciate ligament reconstruction, tendon-bone healing

## Abstract

The objective of this study was to investigate whether intermittent administration of parathyroid hormone [1–34] (PTH[1–34]) promotes tendon-bone healing after anterior cruciate ligament (ACL) reconstruction *in vivo*. A rat model of ACL reconstruction with autograft was established at the left hind leg. Every day, injections of 60 μg PTH[1–34]/kg subcutaneously were given to the PTH group rats (*n* = 10) for four weeks, and the controls (*n* = 10) received saline. The tendon-bone healing process was evaluated by micro-CT, biomechanical test, histological and immunohistochemical analyses. The effects of PTH[1–34] on serum chemistry, bone microarchitecture and expression of the PTH receptor (PTH1R) and osteocalcin were determined. Administration of PTH[1–34] significantly increased serum levels of calcium, alkaline phosphatase (AP), osteocalcin and tartrate-resistant acid phosphatase (TRAP). The expression of PTH1R on both osteocytes and chondrocyte-like cells at the tendon-bone interface was increased in the PTH group. PTH[1–34] also enhanced the thickness and microarchitecture of trabecular bone according to the micro-CT analysis. The results imply that systematically intermittent administration of PTH[1–34] promotes tendon-bone healing at an early stage via up-regulated PTH1R. This method may enable a new strategy for the promotion of tendon-bone healing after ACL reconstruction.

## 1. Introduction

The anterior cruciate ligament (ACL) is an important intra-articular ligament in maintaining the stability and normal motion of the knee joint. ACL injury is a common knee-related injury in the orthopedic surgery department, which influences sport activities, especially in young athletes. It was estimated that over 100,000 ACL reconstructions were performed in the United States each year [1]. ACL reconstruction is the golden standard for the treatment of ACL injury, because of its positive results. Due to reduced donor-site complications compared to bone-patellar tendon-bone graft, the hamstring tendon grafts have been widely used in ACL reconstructions [2,3,4], which are inserted into bone tunnels in the tibia and femur and are anchored at each end by a variety of methods. However, the healing process after ACL reconstruction with hamstring tendon graft, which takes nearly 9 to 12 months, is significantly slower than bone-patellar tendon healing [5,6,7].

ACL reconstruction creates a new tendon-bone interface along the bone tunnel. A successful surgery largely depends on the biological healing at the interface between the tendon graft and the bone tunnel. The fixation site of the tendon within the bone tunnels is the weakest point in the reconstructed knee [8]. Therefore, how to improve tendon-bone healing for functional exercises, daily activities, even sports as early as possible has already become the focus of the field of study. The tendon-bone junction heals slowly due to the bone loss at the site of injury and the avascularity of the fibrocartilage zone [9]. The native direct tendon-bone interface with four typical tissue zones is not reformed during healing, but rather a structurally and mechanically inferior interface [10]. This deficit of microarchitecture in the insertion site raises the subsequent risk of failure after reconstruction surgery [11]. During the past several years, strategies to accelerate and promote tendon-bone healing have been studied in the field of orthopedic basic science.

Parathyroid hormone (PTH) is an anabolic endocrine regulator of calcium and phosphorus homeostasis, and its actions on osteocytes and chondrocytes are mediated by the PTH receptor (PTH1R) [12,13,14]. PTH can activate bone lining cells, prevent osteoblast apoptosis and enhance bone resorption *in vivo* [15]. Recombinant human PTH[1–34], or teriparatide, was approved by the FDA in 2002 for the therapy of osteoporosis in men with high risk of fracture and postmenopausal women [16]. Intermittent PTH[1–34] injections greatly stimulate bone formation and resorption simultaneously, with a greater effect on bone formation than resorption, leading to a net bone gain [17]. PTH[1–34] demonstrates its effects on bone formation by increasing bone volume, connectivity and microarchitecture [18,19]. Evidence from animal model studies has shown that factors that positively impact bone formation and fracture healing, such as osteoinductive growth factors (bone morphogenetic protein (BMP) [20], transforming growth factor (TGF) [21], granulocyte colony-stimulating factor (G-CSF) [22]), osteoconductive materials (injectable calcium phosphate [23], magnesium-based bone adhesives [11]) and low-intensity pulsed ultrasound [24], also positively affect tendon-bone healing [25]. Several studies suggested that intermittently administered PTH[1–34] can promote the remolding and rebuilding process of fracture healing [26,27]. However, its effect on the tendon-bone healing process after ACL reconstruction has not been evaluated.

In this study, we attempted to determine whether PTH[1–34] could be used to improve tendon-bone healing after ACL reconstruction. Firstly, an ACL reconstruction animal model with autograft was established. PTH[1–34] was intermittently administered after surgery for four weeks. We found that daily injections of PTH[1–34] could improve tendon-bone healing, which was proven by micro-CT, biomechanical test and histological assessment.

## 2. Results and Discussion

### 2.1. Results

#### 2.1.1. Serum Chemistry

After four weeks of PTH[1–34] administration, serum levels for calcium, alkaline phosphatase (AP), osteocalcin and tartrate-resistant acid phosphatase (TRAP) were significantly increased in the PTH group compared with the controls (Table 1).

**Table 1:** Serum chemistry (mean ± SD). PTH, parathyroid hormone; AP, alkaline phosphatase; TRAP, tartrate-resistant acid phosphatase; * indicates significant difference between groups.

Concentration	Control	PTH	*p*-Value
Calcium (mmol/L)	2.026 ± 0.396	2.383 ± 0.074 *	0.012
AP (U/L)	180.6 ± 44.7	228.5 ± 22.8 *	0.007
Osteocalcin (pg/mL)	353.07 ± 29.62	425.77 ± 15.98 *	0.000
TRAP (mIU/mL)	2.567 ± 0.892	2.938 ± 0.139 *	0.000

#### 2.1.2. Micro-CT

The averages of the bone tunnel area in the PTH group were significantly smaller than those in the control group (PTH, 2.35 ± 0.38 mm^2^ *vs.* control, 2.88 ± 0.32 mm^2^; *p* = 0.043, *n* = 5). Compared to controls, PTH[1–34] application significantly increased the bone mineral density (BMD), bone volume fraction (BV/TV), bone surface/volume ratio (BS/BV) and trabecular thickness (Tb.Th) of the area we selected. A significant decrease of trabecular separation (Tb.Sp) in the PTH group was observed. No differences in bone surface density (BS/TV) and trabecular number (Tb.N) were detected between the two groups (Table 2).

**Table 2:** Micro-CT evaluation (mean ± SD). BMD, bone mineral density; BV, bone volume; BS, bone surface; Tb.Th, trabecular thickness; Tb.N, trabecular number; Tb.Sp, trabecular separation; * indicates significant difference between groups.

Items	Control	PTH	*p-*Value
Area (mm^2^)	2.88 ± 0.32	2.35 ± 0.38 *	0.043
BMD (mg/mL)	0.3426 ± 0.0059	0.4009 ± 0.0083 *	0.000
BV/TV (%)	28.52 ± 1.56	33.43 ± 1.67 *	0.001
BS/BV (1/mm)	11.64225 ± 1.369840	16.24998 ± 1.580501 *	0.001
BS/TV (1/mm)	3.900066 ± 0.574287	4.625814 ± 0.652808	0.099
Tb.Th (mm)	0.235796 ± 0.053191	0.349876 ± 0.071286 *	0.021
Tb.N (1/mm)	0.992012 ± 0.233659	1.243782 ± 0.260488	0.146
Tb.Sp (mm)	1.061898 ± 0.099597	0.81308 ± 0.144448 *	0.013

#### 2.1.3. Biomechanical Test

As the evaluation of failure load, all grafts in both groups failed by total pullout from the bone tunnels. The average of failure load in the PTH group was significantly greater than that in the control group at four weeks after operation (PTH, 16.49 ± 4.90 N *vs.* control, 8.17 ± 0.59 N; *p* = 0.019, *n* = 5). There was no significant difference in stiffness between the two groups (PTH, 4.32 ± 1.95 N/mm *vs.* control, 2.48 ± 0.47 N/mm; *p* = 0.102, *n* = 5; Table 3).

**Table 3:** Biomechanical test (mean ± SD). * indicates significant difference between groups.

Items	Control	PTH	*p-*Value
Failure load (N)	8.17 ± 0.59	16.49 ± 4.90 *	0.005
Stiffness (N/mm)	2.48 ± 0.47	4.32 ± 1.95	0.102

#### 2.1.4. Histological Staining

**Figure 1:**
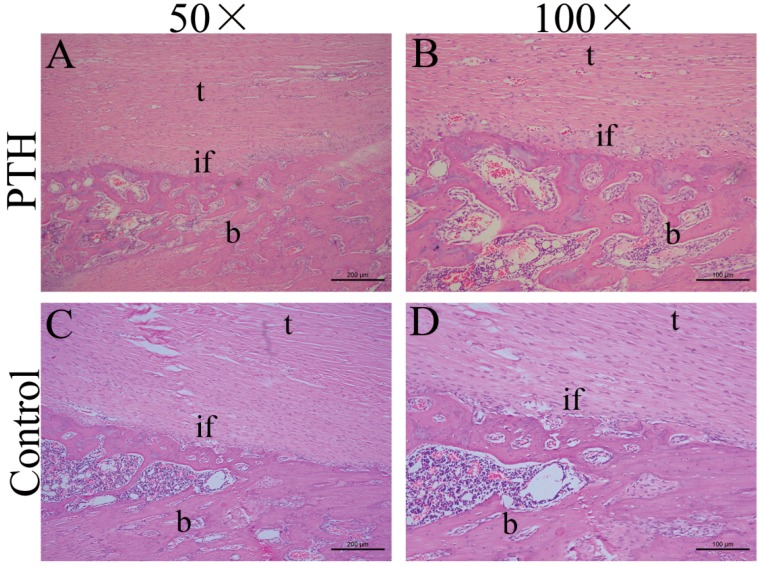
Histological observation of the tendon-bone interface in the PTH group (**A**,**B**) and the control group (**C**,**D**) by hematoxylin and eosin (H&E) staining (t, tendon; b, bone; if, interface).

**Figure 2:**
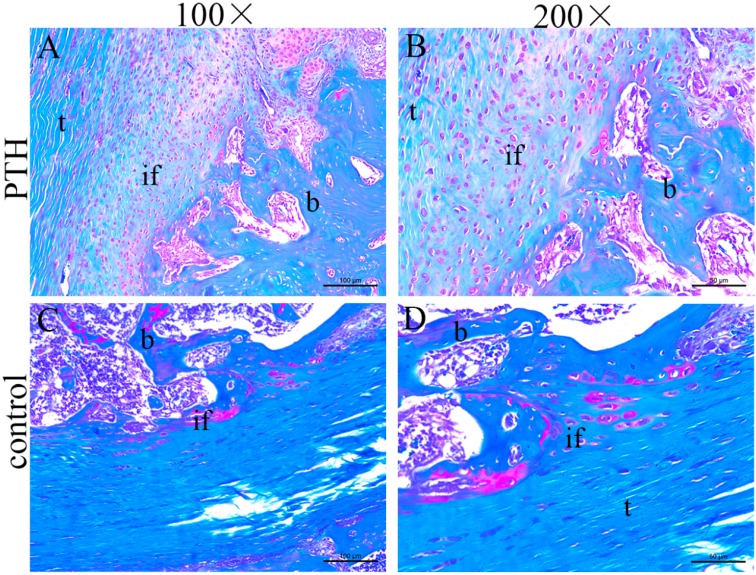
Histological observation of the tendon-bone interface in the PTH group (**A**,**B**) and the control group (**C**,**D**) by Safranin O staining (t, tendon; b, bone; if, interface).

In the two groups, the fibrous connective tissue was aligned, and the tendon-bone interface was covered by the woven bone. In the PTH group, there were more aligned chondrocyte-like cells than in the control group. At the interface of the control group, chondrocyte-like cells were barely observed (Figure 1). Staining with Safranin O showed larger collection of cartilage cells and more abundant proteoglycan production at the tendon-bone interface in the PTH group than in the control group. However, these cartilage cells were immature, and the typical four histological transitions were not observed (Figure 2).

#### 2.1.5. Immunohistochemistry Staining

**Figure 3:**
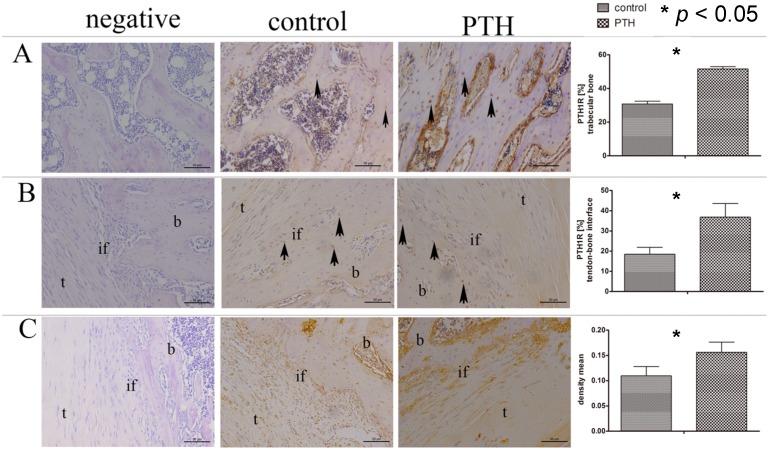
PTH1R expression in the trabecular bone (**A**) and tendon-bone interface (**B**) was up-regulated following PTH[1–34] administration. The arrows (**A**,**B**) point at some of the positive stained cells; (**C**) The density mean of immunoreactivity to osteocalcin at the tendon-bone interface of the PTH group was higher than that of the control group. Negative controls (left images in **A**–**C**) show immunostaining with the omission of primary antibody. Scale bar: 50 µm.

After administration of PTH[1–34] for four weeks, a consecutive up-regulation of PTH1R expression on both osteocytes in the trabecular bone and chondrocyte-like cells at the tendon-bone interface was observed. Immunoreactivity to osteocalcin of the PTH group was significantly higher than that of the control group (Figure 3).

### 2.2. Discussion

In the present study, we elucidated the effectiveness of PTH[1–34], which was intermittently administered after operation for early tendon-bone healing in ACL reconstruction using a rat model. Radiological, biomechanical and histological assessment at the site of the tendon-bone interface demonstrated the promoted healing in the PTH group. We noted that application of PTH[1–34] directly affected the cells mediating tendon-bone healing, as PTH1R expression was significantly up-regulated at the tendon-bone interface. The study demonstrated a therapeutic strategy using PTH[1–34] for tendon-bone healing after ACL reconstruction.

The dosages of PTH[1–34] are various among different experimental investigations [28]. In our study, 60 μg PTH[1–34]/kg/day was chosen, because it was the median among several investigations in which bone formation was improved in a rat model [29,30]. Intermittent and short-term administration of PTH[1–34] could favor bone formation in animals [31] and human [32], and high levels of dosage induced a significant increase in serum calcium and AP concentration [33]. PTH not only strongly stimulates bone formation during the bone remodeling process, but also promotes bone resorption [34]. In our present study, a significant increase in the serum concentration of osteocalcin and TRAP was observed.

PTH[1–34] increases BMD, BV/TV and connectivity, enhancing bone strength and reducing the fracture risk in the animal models and human patients [28,32,35]. Bellido *et al.* [36] found that PTH[1–34] increased the volume and thickness of cortical and trabecular bone through evaluation of bone microarchitecture by micro-CT. These results fit in with the present study in which PTH[1–34] exerted its effects on BMD, BV/TV, BS/TV and Tb.Sp at the tendon-bone interface evaluated by micro-CT. Remarkably, the impact on the bone microarchitecture was accompanied by the increase in serum levels of calcium, AP, osteocalcin and TRAP. Accordingly, much more chondrocyte-like cells were observed at the tendon-bone interface in the PTH group than in the control group under microscopy.

Solid tendon-bone healing is a prerequisite for successful ACL reconstruction [37]. In our present study, the failure load in the PTH group was larger than that in the control group, but there was no significant difference in stiffness between the two groups. This was because the remolding and rebuilding of the tendon-bone interface was just at an early phase when failures were generally via graft pullout [38]. The chondral tendon-bone healing was observed more in the PTH group, but incompletely matured chondral healing could not strengthen the biomechanical properties effectively [39].

The cell types that initiate and regulate the tendon-bone healing process have not been concretely identified. It has been reported that in the first two weeks following ACL reconstruction, graft cells do not survive and the graft undergoes necrosis [40]. It seems that the host cells from the surrounding bone marrow, which contains bone mesenchymal stem cells (BMSCs) rather than graft cells, contribute to the repair of the tendon-bone interface [40,41]. Several studies suggested that MSCs could promote tendon-bone healing after ACL reconstruction. Lim *et al.* [42] used hamstring tendon autograft coated with MSCs to reconstruct ACL in adult rabbits and found that MSC-enhanced grafts had significantly higher failure loads and stiffness at eight weeks after surgery. Soon *et al.* [43] found that the application of MSCs at the allograft tendon-bone interface during ACL reconstruction could develop an intervening zone of fibrocartilage. PTH1R is the only receptor for PTH in bone tissue and is expressed in osteoblast lineage cells, including BMSCs [44], osteoblasts [45] and osteocytes [46]. During the tendon-bone healing process, PTH could bind to PTH1R in MSCs and osteoblasts and accelerate the tendon-bone healing. Chen *et al.* [47] demonstrated that PTH could increase the fluorescence intensity of calcium ions and the proliferation of MSC *in vitro*. Alternatively, PTH could affect the tendon-bone healing process through regulating the secretion of certain factors from MSCs, osteocytes or osteoblasts. The significant up-regulation of PTH1R on osteocytes and chondrocytes at the tendon-bone interface indicated that systemic application of PTH[1–34] was capable of directly stimulating these cells. Chondrocytes and osteocytes express PTH1R [12,14], and binding of PTH[1–34] to the receptor may enhance the expression and production of growth factors [48,49]. In our present study, the coexistent increase in BMD, BV/TV and the improvement of tendon-bone healing most likely is ascribed to the up-regulated PTH1R expression on osteocytes and chondrocyte-like cells. Osteocalcin is a marker of terminal osteoblast differentiation and influences bone mineralization. In the PTH group, higher osteocalcin density indicated the positive effectiveness of PTH on osteogenesis. However, more studies are needed to make the underlying mechanisms clear.

There are some limitations in our study. Our specimens were harvested at only one time point (four weeks) postoperatively. The long-term effect of PTH on tendon-bone healing could not be observed. In a future study, the long-term effect of the administration of PTH will be investigated.

## 3. Experimental Section

### 3.1. Study Design

Twenty male Sprague Dawley rats (12 weeks of age) were used in this study. Animals were allocated to 2 equal numbered groups randomly (control group and PTH group), and surgery was performed. Animals of the control group and PTH group received vehicle and recombinant PTH[1–34] (60 μg/kg/day, Bachem, Bubendorf, Switzerland), respectively, daily from Day 1 to 4 weeks postoperatively. At 4 weeks postoperatively, all animals were euthanized, and the blood and tibia–tendon–femur complex of the left hind leg were harvested for analyses. The study protocol was approved by the Zhejiang University Institutional Animal Care and Use Committee (Register ID No.: ZJU2014032006; Date: 6 May 2014).

### 3.2. Surgical Procedure

**Figure 4:**
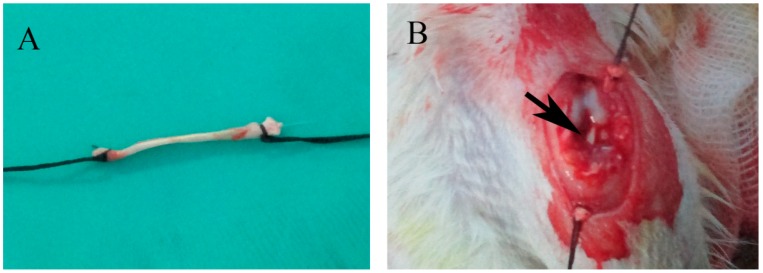
(**A**) Gross observation of the flexor digitorum longus tendon tied with 4–0 sutures at the two ends; (**B**) Macroscopic view of anterior cruciate ligament (ACL) reconstruction (the arrow points to the flex digitorum longus tendon).

A unilateral rat ACL reconstruction model was adopted as described previously [41] and slightly modified [50]. The surgical procedure was performed under strictly aseptic conditions. After general anesthesia with an intraperitoneal injection of pentobarbital sodium solution (Kyoritsu-seiyaku, 50 mg/kg body weight), a 2-cm skin incision was made just posterior to the ankle and ending at the medial and palmar side of the first metatarsal at the right hind leg. The flexor digitorum longus tendon was identified and cut transversely at the midmetatarsal level. The full length of the tendon was about 4 cm after being stripped from the posterior tibia, and then, both ends were sutured using 4–0 Ethibond (Ethicon, Somerville, NJ, USA). A 2-cm longitudinal skin incision was used to expose the left knee joint. The patella was dislocated medially, and then, the native ACL was excised. The tibial and femoral bone tunnels were created with a 1.2-mm diameter Kirschner wire (K-wire; Zimmer, Warswa, IN, USA). The full length of the bone tunnel from anteromedial tibia, across the joint, to the anterolateral femur was about 20 mm. The flexor digitorum longus tendon was passed through the tunnel, and both ends were sutured to the periosteum and surrounding soft tissue using the 4–0 Ethibond suture (Figure 4). The animals were then returned to their cages and allowed to move freely.

### 3.3. Serum Chemistry

Blood was collected from aorta abdominalis at the time of euthanization. Serum was separated with centrifugation at 3000 r/min for 10 min. Serum levels of calcium and alkaline phosphatase (AP) were analyzed by the automatic biochemical analyzer (Beckman Coulter Au5400, Beckman Coulter Inc., Brea, CA, USA). Serum osteocalcin and TRAP level were analyzed by a Rat Osteocalcin ELISA Kit (Rapidbio Inc., Los Angeles, CA, USA) and a Rat TRAP ELISA Kit (Rapidbio).

### 3.4. Micro-CT

**Figure 5:**
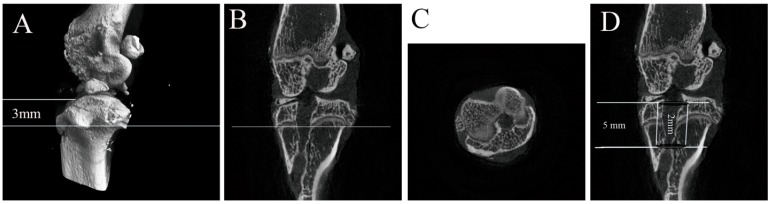
A sketch map of micro-CT evaluations: (**A**) observation of the vertical plane of the axis of the bone tunnel at a 3-mm depth from the tibial joint surface in a three-dimensional reconstruction micro-CT image; (**B**) the vertical plane of the axis of the bone tunnel at a 3-mm depth from the tibial joint surface in a sagittal view of the micro-CT image; (**C**) the areas of the bone tunnel at a 3-mm depth from the tibial joint were measured with Image-Pro Plus; (**D**) BMD, BV/TV, BS/BV, BS/TV, Tb.Th, Tb.N and Tb.Sp of a cylinder scope, including the bone tunnel with a 2.0-mm diameter and depth of 5.0-mm from the joint surface, were calculated.

The left knee joints (*n* = 5 for each group) were collected at 4 weeks postoperatively and kept at −80 °C immediately. Before testing, the specimens were thawed overnight at 4 °C. Gross observation and radiographs were used to determine the mineralized tissue formation in bone tunnels and the areas of the vertical plane of the axis of the bone tunnel at 3-mm inferior to the tibial joint surface (micro-CT, 36-μm thickness; Skyscan1176, BRUKER, Antwerp, Belgium). Each area was measured an average of three times with Image-Pro Plus 6.0 software (IPP 6.0, Media Cybernetics Inc., Rockville, MD, USA). The trabecular bone mineral density (BMD), bone volume fraction (BV/TV), bone surface/volume ratio (BS/BV), bone surface density (BS/TV), trabecular thickness (Tb.Th), trabecular number (Tb.N) and trabecular separation (Tb.Sp) of a cylinder scope, including the bone tunnel with a 2.0-mm diameter and depth of 5.0 mm from the joint surface were calculated by 3D standard microstructural analysis (Figure 5) [51].

### 3.5. Biomechanical Test

After micro-CT assessment, the biomechanical test was performed (*n* = 5 for each group). All soft tissue, except for the ACL graft, was dissected to create a femur–graft–tibia complex. The tibia and femur were embedded in 15 mL centrifugal tubes with dental cement. Additionally, the two ends of the complex were fixed in the tailor-made cylinder with screws. The cylinders were clamped in an Instron 553A material testing system (Instron, Boston, MA, USA) at 45° flexion. The biomechanical test was performed by increasing the tensile load with a crosshead speed of 5 mm/min. The failure load (N) and elongation were recorded, and the stiffness (N/mm) was calculated from the slop of the load–deformation curve. The specimens were kept always moist with normal saline (NS).

### 3.6. Histological and Immunohistochemical Analyses

The specimens (*n* = 5 for each group) were harvested and fixed in 4% paraformaldehyde for 48 h and then proceeded to decalcification in 10% ethylenediaminetetraacetic acid (EDTA) with 0.1 M phosphate-buffered saline (PBS) for 3 weeks. After the dehydration with a graded alcohol solution and being embedded in paraffin wax, the specimens were sectioned parallel to the longitudinal axis of the bone tunnel. Sections were cut at 5 μm and stained with hematoxylin and eosin (H&E staining) for traditional light microscopy. Safranin O staining was also performed to observe the fibrocartilage formation in the tendon-bone interface with light microscopy.

Prior to application of primary antibodies, the Ultra V Block (Lab Vision Corporation, Fremont, CA, USA) was used to incubate in sections to reduce the non-specific background staining. For immunohistochemistry staining, monoclonal antibodies for PTH1R (Abcam, Cambridge, UK) and osteocalcin (Abcam) were applied for 12 h at 4 °C. The incubation with biotinylated goat anti-rabbit antibody (Lab Vision Corporation) for 1 h at room temperature was followed. Streptavidin peroxidase was applied for 45 min, and 3,3'-diaminobenzidine was added as a chromogenic agent. Counterstaining was done with hematoxylin, and the negative control was set by PBS instead of the primary antibodies.

For the evaluation of PTH1R immunostaining, histological section images taken at 200-fold were digitalized, and five standardized regions of interest (ROIs) were defined at the trabecular bone and tendon-bone interface. PTH1R expression was determined as the percentage of positive stained cells in the standardized ROIs. Immunoreactivity to osteocalcin in the tendon-bone interface of PTH group was compared with that of the control group. The ROI was defined as the area of the whole image selected. The integrated option density (IOD) and the density mean (IOD/ROI) were analyzed by the Image-Pro Plus 6.0 software (IPP 6.0)

### 3.7. Statistical Analysis

The data were expressed as the mean ± standard deviation (SD). All data were analyzed using SPSS 16.0 software (SPSS, Chicago, IL, USA), and *p* < 0.05 was considered statistically significant. An independent *t*-test or nonparametric Wilcoxon–Mann–Whiney test was used to detect the difference between the two groups.

## 4. Conclusions

In conclusion, the systematically intermittent administration of PTH[1–34] promotes tendon-bone healing at an early stage. This method may enable a new strategy for the promotion of tendon-bone healing after ACL reconstruction.

## References

[1] Harner C.D., Giffin J.R., Dunteman R.C., Annunziata C.C., Friedman M.J. (2001). Evaluation and treatment of recurrent instability after anterior cruciate ligament reconstruction. Instr. Course Lect..

[2] Fujita N., Kuroda R., Matsumoto T., Yamaguchi M., Yagi M., Matsumoto A., Kubo S., Matsushita T., Hoshino Y., Nishimoto K. (2011). Comparison of the clinical outcome of double-bundle, anteromedial single-bundle, and posterolateral single-bundle anterior cruciate ligament reconstruction using hamstring tendon graft with minimum 2-year follow-up. Arthroscopy.

[3] Giron F., Cuomo P., Edwards A., Bull A.M., Amis A.A., Aglietti P. (2007). Double-bundle “anatomic” anterior cruciate ligament reconstruction: A cadaveric study of tunnel positioning with a transtibial technique. Arthroscopy.

[4] Fu F.H., Shen W., Starman J.S., Okeke N., Irrgang J.J. (2008). Primary anatomic double-bundle anterior cruciate ligament reconstruction: A preliminary 2-year prospective study. Am. J. Sports Med..

[5] Tomita F., Yasuda K., Mikami S., Sakai T., Yamazaki S., Tohyama H. (2001). Comparisons of intraosseous graft healing between the doubled flexor tendon graft and the bone-patellar tendon-bone graft in anterior cruciate ligament reconstruction. Arthroscopy.

[6] Park M.J., Lee M.C., Seong S.C. (2001). A comparative study of the healing of tendon autograft and tendon-bone autograft using patellar tendon in rabbits. Int. Orthop..

[7] Papageorgiou C.D., Ma C.B., Abramowitch S.D., Clineff T.D., Woo S.L. (2001). A multidisciplinary study of the healing of an intraarticular anterior cruciate ligament graft in a goat model. Am. J. Sports Med..

[8] Rodeo S.A., Suzuki K., Deng X.H., Wozney J., Warren R.F. (1999). Use of recombinant human bone morphogenetic protein-2 to enhance tendon healing in a bone tunnel. Am. J. Sports Med..

[9] Wong M.W., Qin L., Tai J.K., Lee S.K., Leung K.S., Chan K.M. (2004). Engineered allogeneic chondrocyte pellet for reconstruction of fibrocartilage zone at bone-tendon junction—A preliminary histological observation. J. Biomed. Mater. Res. B.

[10] Rothrauff B.B., Tuan R.S. (2014). Cellular therapy in bone-tendon interface regeneration. Organogenesis.

[11] Gulotta L.V., Kovacevic D., Ying L., Ehteshami J.R., Montgomery S., Rodeo S.A. (2008). Augmentation of tendon-to-bone healing with a magnesium-based bone adhesive. Am. J. Sports Med..

[12] Rhee Y., Allen M.R., Condon K., Lezcano V., Ronda A.C., Galli C., Olivos N., Passeri G., O’Brien C.A., Bivi N. (2011). PTH receptor signaling in osteocytes governs periosteal bone formation and intracortical remodeling. J. Bone Miner. Res..

[13] Bukata S.V. (2011). Systemic administration of pharmacological agents and bone repair: What can we expect. Injury.

[14] Weisser J., Riemer S., Schmidl M., Suva L.J., Poschl E., Brauer R., von der Mark K. (2002). Four distinct chondrocyte populations in the fetal bovine growth plate: Highest expression levels of PTH/PTHrP receptor, Indian hedgehog, and MMP-13 in hypertrophic chondrocytes and their suppression by PTH (1–34) and PTHrP (1–40). Exp. Cell Res..

[15] Jilka R.L. (2007). Molecular and cellular mechanisms of the anabolic effect of intermittent PTH. Bone.

[16] Chandra A., Lan S., Zhu J., Lin T., Zhang X., Siclari V.A., Altman A.R., Cengel K.A., Liu X.S., Qin L. (2013). PTH prevents the adverse effects of focal radiation on bone architecture in young rats. Bone.

[17] Qin L., Raggatt L.J., Partridge N.C. (2004). Parathyroid hormone: A double-edged sword for bone metabolism. Trends Endocrinol. Metab..

[18] Recker R.R., Bare S.P., Smith S.Y., Varela A., Miller M.A., Morris S.A., Fox J. (2009). Cancellous and cortical bone architecture and turnover at the iliac crest of postmenopausal osteoporotic women treated with parathyroid hormone 1–84. Bone.

[19] Jiang Y., Zhao J.J., Mitlak B.H., Wang O., Genant H.K., Eriksen E.F. (2003). Recombinant human parathyroid hormone (1–34) [teriparatide] improves both cortical and cancellous bone structure. J. Bone Miner. Res..

[20] Ma C.B., Kawamura S., Deng X.H., Ying L., Schneidkraut J., Hays P., Rodeo S.A. (2007). Bone morphogenetic proteins-signaling plays a role in tendon-to-bone healing: A study of rhBMP-2 and noggin. Am. J. Sports Med..

[21] Manning C.N., Kim H.M., Sakiyama-Elbert S., Galatz L.M., Havlioglu N., Thomopoulos S. (2011). Sustained delivery of transforming growth factor beta three enhances tendon-to-bone healing in a rat model. J. Orthop. Res..

[22] Sasaki K., Kuroda R., Ishida K., Kubo S., Matsumoto T., Mifune Y., Kinoshita K., Tei K., Akisue T., Tabata Y. (2008). Enhancement of tendon-bone osteointegration of anterior cruciate ligament graft using granulocyte colony-stimulating factor. Am. J. Sports Med..

[23] Kovacevic D., Fox A.J., Bedi A., Ying L., Deng X.H., Warren R.F., Rodeo S.A. (2011). Calcium–phosphate matrix with or without TGF-β3 improves tendon-bone healing after rotator cuff repair. Am. J. Sports Med..

[24] Walsh W.R., Stephens P., Vizesi F., Bruce W., Huckle J., Yu Y. (2007). Effects of low-intensity pulsed ultrasound on tendon-bone healing in an intra-articular sheep knee model. Arthroscopy.

[25] Atesok K., Fu F.H., Wolf M.R., Ochi M., Jazrawi L.M., Doral M.N., Lubowitz J.H., Rodeo S.A. (2014). Augmentation of tendon-to-bone healing. J. Bone Jt. Surg. Am..

[26] Ellegaard M., Kringelbach T., Syberg S., Petersen S., Beck Jensen J.E., Bruel A., Jorgensen N.R., Schwarz P. (2013). The effect of PTH(1–34) on fracture healing during different loading conditions. J. Bone Miner. Res..

[27] Malhotra R., Meena S., Digge V.K. (2013). Tensile type of stress fracture neck of femur: Role of teriparatide in the process of healing in a high risk patient for impaired healing of fracture. Clin. Cases Mineral Bone Metab..

[28] Ellegaard M., Jorgensen N.R., Schwarz P. (2010). Parathyroid hormone and bone healing. Calcif. Tissue Int..

[29] Bruel A., Vegger J.B., Raffalt A.C., Andersen J.E., Thomsen J.S. (2013). PTH(1–34), but not strontium ranelate counteract loss of trabecular thickness and bone strength in disuse osteopenic rats. Bone.

[30] Li Y.F., Li X.D., Bao C.Y., Chen Q.M., Zhang H., Hu J. (2013). Promotion of peri-implant bone healing by systemically administered parathyroid hormone (1–34) and zoledronic acid adsorbed onto the implant surface. Osteoporos. Int..

[31] Hock J.M., Gera I. (1992). Effects of continuous and intermittent administration and inhibition of resorption on the anabolic response of bone to parathyroid hormone. J. Bone Miner. Res..

[32] Neer R.M., Arnaud C.D., Zanchetta J.R., Prince R., Gaich G.A., Reginster J.Y., Hodsman A.B., Eriksen E.F., Ish-Shalom S., Genant H.K. (2001). Effect of parathyroid hormone (1–34) on fractures and bone mineral density in postmenopausal women with osteoporosis. N. Engl. J. Med..

[33] Hirano T., Burr D.B., Turner C.H., Sato M., Cain R.L., Hock J.M. (1999). Anabolic effects of human biosynthetic parathyroid hormone fragment (1–34), LY333334, on remodeling and mechanical properties of cortical bone in rabbits. J. Bone Miner. Res..

[34] Wu X., Pang L., Lei W., Lu W., Li J., Li Z., Frassica F.J., Chen X., Wan M., Cao X. (2010). Inhibition of Sca-1-positive skeletal stem cell recruitment by alendronate blunts the anabolic effects of parathyroid hormone on bone remodeling. Cell Stem Cell.

[35] Brouwers J.E., van Rietbergen B., Huiskes R., Ito K. (2009). Effects of PTH treatment on tibial bone of ovariectomized rats assessed by *in vivo* micro-CT. Osteoporos. Int..

[36] Bellido M., Lugo L., Roman-Blas J.A., Castaneda S., Calvo E., Largo R., Herrero-Beaumont G. (2011). Improving subchondral bone integrity reduces progression of cartilage damage in experimental osteoarthritis preceded by osteoporosis. Osteoarthr. Cartil..

[37] Fan H., Liu H., Wong E.J., Toh S.L., Goh J.C. (2008). *In vivo* study of anterior cruciate ligament regeneration using mesenchymal stem cells and silk scaffold. Biomaterials.

[38] Pinczewski L.A., Clingeleffer A.J., Otto D.D., Bonar S.F., Corry I.S. (1997). Integration of hamstring tendon graft with bone in reconstruction of the anterior cruciate ligament. Arthroscopy.

[39] Oka S., Matsumoto T., Kubo S., Matsushita T., Sasaki H., Nishizawa Y., Matsuzaki T., Saito T., Nishida K., Tabata Y. (2013). Local administration of low-dose simvastatin-conjugated gelatin hydrogel for tendon-bone healing in anterior cruciate ligament reconstruction. Tissue Eng. A.

[40] Kobayashi M., Watanabe N., Oshima Y., Kajikawa Y., Kawata M., Kubo T. (2005). The fate of host and graft cells in early healing of bone tunnel after tendon graft. Am. J. Sports Med..

[41] Kawamura S., Ying L., Kim H., Dynybil C., Rodeo S. (2005). Macrophages accumulate in the early phase of tendon-bone healing. J. Orthop. Res..

[42] Lim J.K., Hui J., Li L., Thambyah A., Goh J., Lee E.H. (2004). Enhancement of tendon graft osteointegration using mesenchymal stem cells in a rabbit model of anterior cruciate ligament reconstruction. Arthroscopy.

[43] Soon M.Y., Hassan A., Hui J.H., Goh J.C., Lee E.H. (2007). An analysis of soft tissue allograft anterior cruciate ligament reconstruction in a rabbit model: A short-term study of the use of mesenchymal stem cells to enhance tendon osteointegration. Am. J. Sports Med..

[44] Mendez-Ferrer S., Michurina T.V., Ferraro F., Mazloom A.R., Macarthur B.D., Lira S.A., Scadden D.T., Ma’ayan A., Enikolopov G.N., Frenette P.S. (2010). Mesenchymal and haematopoietic stem cells form a unique bone marrow niche. Nature.

[45] Fermor B., Skerry T.M. (1995). PTH/PTHrP receptor expression on osteoblasts and osteocytes but not resorbing bone surfaces in growing rats. J. Bone Miner. Res..

[46] Bellido T., Saini V., Pajevic P.D. (2013). Effects of PTH on osteocyte function. Bone.

[47] Chen Y., Bai B., Zhang S., Ye J., Chen Y., Zeng Y. (2014). Effects of parathyroid hormone on calcium ions in rat bone marrow mesenchymal stem cells. BioMed Res. Int..

[48] Coleman D.T., Bilezikian J.P. (1990). Parathyroid hormone stimulates formation of inositol phosphates in a membrane preparation of canine renal cortical tubular cells. J. Bone Miner. Res..

[49] Wang Y., Nishida S., Boudignon B.M., Burghardt A., Elalieh H.Z., Hamilton M.M., Majumdar S., Halloran B.P., Clemens T.L., Bikle D.D. (2007). IGF-I receptor is required for the anabolic actions of parathyroid hormone on bone. J. Bone Miner. Res..

[50] Lovric V., Chen D., Yu Y., Oliver R.A., Genin F., Walsh W.R. (2012). Effects of demineralized bone matrix on tendon-bone healing in an intra-articular rodent model. Am. J. Sports Med..

[51] Bouxsein M.L., Boyd S.K., Christiansen B.A., Guldberg R.E., Jepsen K.J., Muller R. (2010). Guidelines for assessment of bone microstructure in rodents using micro-computed tomography. J. Bone Miner. Res..

